# T Cell Epitope Mapping of the E-Protein of West Nile Virus in BALB/c Mice

**DOI:** 10.1371/journal.pone.0115343

**Published:** 2014-12-15

**Authors:** Marina De Filette, Stefan Chabierski, Oliwia Andries, Sebastian Ulbert, Niek N. Sanders

**Affiliations:** 1 Laboratory of Gene Therapy, Faculty of Veterinary Sciences, Ghent University, Merelbeke, Belgium; 2 Department of Immunology, Fraunhofer Institute for Cell Therapy and Immunology, Leipzig, Germany; Metabiota, United States of America

## Abstract

West Nile virus (WNV) is a zoonotic virus, which is transmitted by mosquitoes. It is the causative agent of the disease syndrome called West Nile fever. In some human cases, a WNV infection can be associated with severe neurological symptoms. The immune response to WNV is multifactorial and includes both humoral and cellular immunity. T-cell epitope mapping of the WNV envelope (E) protein has been performed in C57BL/6 mice, but not in BALB/c mice. Therefore, we performed in BALB/c mice a T-cell epitope mapping using a series of peptides spanning the WNV envelope (E) protein. To this end, the WNV-E specific T cell repertoire was first expanded by vaccinating BALB/c mice with a DNA vaccine that generates subviral particles that resemble West Nile virus. Furthermore, the WNV structural protein was expressed in *Escherichia coli* as a series of overlapping 20-mer peptides fused to a carrier-protein. Cytokine-based ELISPOT assays using these purified peptides revealed positive WNV-specific T cell responses to peptides within the different domains of the E-protein.

## Introduction

West Nile virus (WNV) is a small enveloped single-stranded positive sense RNA-containing virus that belongs to the family *Flaviviridae* [1]. The virus is maintained in an enzootic cycle where it is transmitted between ornithophilic mosquitoes of the *Culex* genus and birds. Equine and humans are considered dead-end hosts since they do not mount high enough viremia for mosquitoes to become infected following feeding [2]. Human infection with the virus leads to a wide range of diseases from mildly febrile to severe neurologic complications and death, but asymptomatic infections occur most frequently [3]. Humoral immunity is considered an essential aspect of protective immunity since it limits WNV dissemination into the nervous system. This was demonstrated in mice lacking B cells which developed high-grade viremia, early dissemination into the brain and uniform mortality [4]. The envelope E glycoprotein is the principal antigen that elicits neutralizing antibodies and as such is a primary target for vaccine development [5]. Studies in animal models have also demonstrated that T lymphocytes are an essential component of protection against WNV. Mice deficient in CD8+ T cells develop persistent WNV infections in the brain [6], [7]. Studies in mice have also shown that CD4+ T cells control WNV infection by priming B cell and antibody responses, and by sustaining CD8+ T cell activity [8]. Mapping of antigenic peptide sequences from proteins of relevant pathogens recognized by T helper (Th) and by cytolytic T lymphocytes (CTL) may help to understand virus immunity and pathogenesis. The majority of T cells recognize peptide epitopes bound to major histocompatibility complex (MHC)-encoded glycoproteins on the surface of professional antigen-presenting cells APC [9]. Most T cells are specific for peptide epitopes in association with either classical MHC class I molecules (H2-K, D, and L in mice) in the case of CD8+ T cells, or class II molecules (H2-A and E in mice) for CD4+ T cells [10]. These peptide antigens are subsequently detected by the T cell receptor of T cells, which proliferate, secrete cytokines and differentiate into antigen-specific effector cells [11], [12]. Nearly all of the epitopes associated with protective responses against WNV using mice models are non-linear and conformational B-cell epitopes and most of these are specific to the envelope (E) protein. The majority of these B-cell epitopes have been defined in BALB/c mice [13] whereas C57BL/6 mice have been used mostly to identify T-cell epitopes [13]–[16]. Both mouse strains are equally susceptible to WNV infection [17], [18] but C57BL/6 mice demonstrate elevated blood-brain barrier permeability [17]. In addition, as C57BL/6 mice predominantly show Th1-dependent immune responses upon infection, whereas BALB/c mice tend to favor Th2-responses, T-cell epitopes identified in one strain might not be directly transferable to the other.

Bioinformatics experts have developed computer-driven algorithms methods to predict T cell epitopes which have significantly decreased the experimental burden that is associated with epitope identification [11], [19]–[21]. To predict in BALB/c mice potential T-cell epitopes of the E-protein of the WNV, we used the Immune Epitope Database and Analysis Resource (IEDB, [22]). To confirm these predictions with *in vivo* immunogenicity, we vaccinated BALB/c mice with a plasmid expressing the membrane protein M (prM) and E protein. Subsequently, we measured the CD8+ and CD4+ T cell cytokine responses using a series of peptides derived from WNV E-protein. As the DNA vaccine leads to the expression of virus-like-particles resembling correctly folded E and M proteins, we can assume that the identified epitopes will also exist during the course of a WNV infection [23].

## Findings

**Figure 1:**
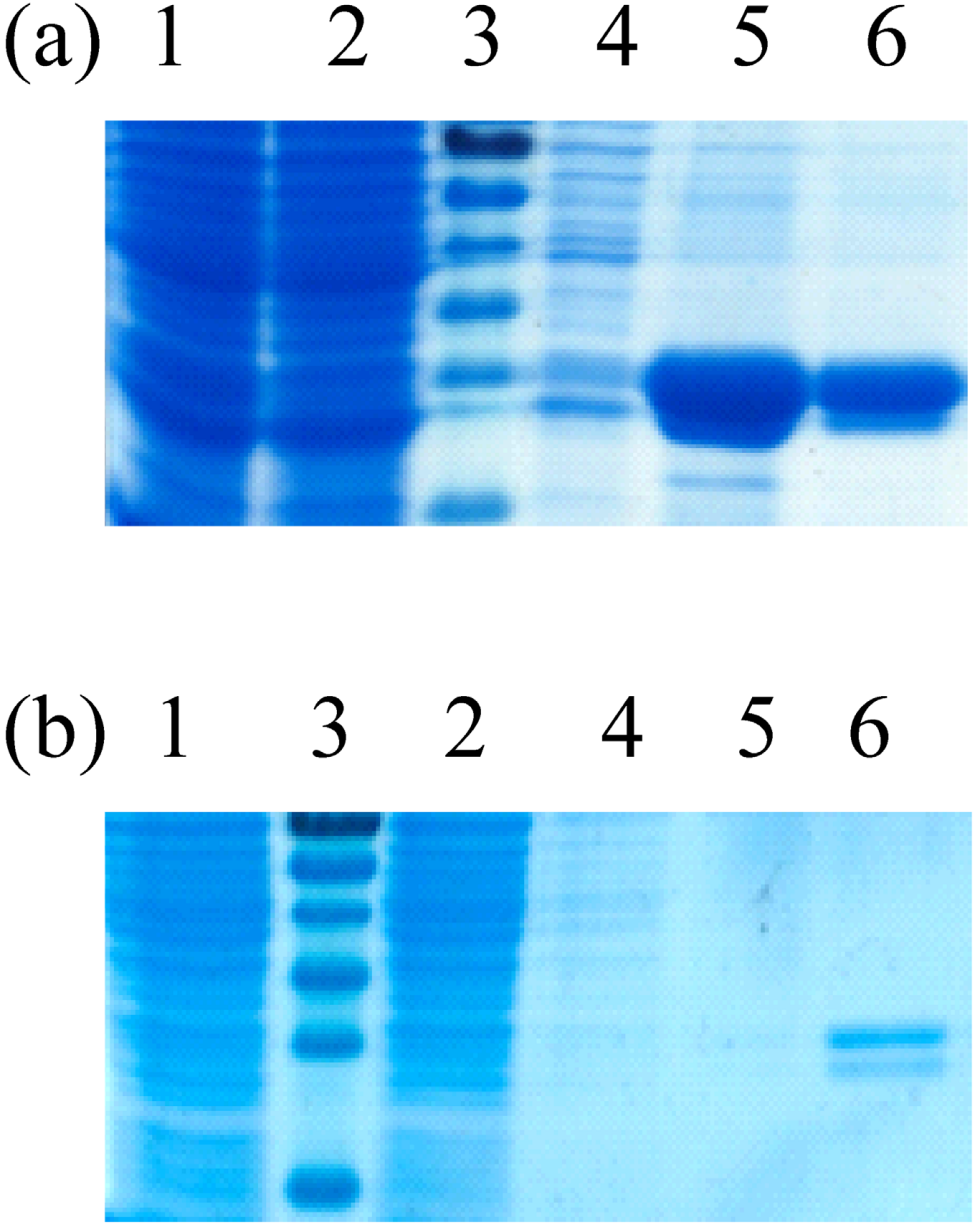
Expression and purification of recombinant GST tagged peptides. SDS-PAGE showing crude lysates of protein-expressing bacteria and purification steps of peptide E471 showing a moderate expression (a) and peptide E131 showing very low expression (b). Lane 1, crude lysate; lane 2, lysate supernatant after centrifugation; lane 3, size marker; lanes 4–5, respectively elution fraction 1 and 2 of recombinant peptide after glutathione affinity purification.

The sequence spanning the E protein of the lineage 1 WNV strain “Ita09” (NCBI Acc#.210 GU011992) was separated into 26 clones, each coding for 30 amino acid long peptides with an overlap of 10 amino acids on both sides. The DNA fragments encoding these peptides were cloned in the bacterial expression vector pEXP1 by ATG:biosynthetics (Freiburg, Germany) after the sequence of glutathione-S-transferase (GST) from *Schistosoma japonicum*. The sequence of the different bacterial expression vectors were sequenced to ensure that they contained the right base pair sequences and hence encoded the desired peptides. After transformation of these vectors into the *E. coli* BL21 strain, selected colonies were grown in Luria Bertani broth containing kanamycine and induced overnight with isopropylthio-β-galactoside (IPTG, 1 mM final concentration, Sigma–Aldrich (Diegem, Belgium)). The bacteria were collected by centrifugation (20 min at 4,000 g at 4°C) and the cell pellet was resuspended in lysis buffer (phosphate buffered saline (PBS, Invitrogen (Merelbeke, Belgium)) containing protease inhibitors cocktail (Roche, Mannheim, Germany), 1 mM DTT, 1 mM EDTA, 1% Triton X-100 and Lysozyme (1 mg/ml)). Lysates were obtained after sonication and were subsequently clarified by centrifugation (30 min at 15,000×g at 4°C). Recombinant peptides derived from the E-protein were purified by glutathione affinity purification and dialyzed against sterile PBS before use. The purified proteins were analyzed in denaturing protein gels and the size difference compared to GST alone was visualized as previously described by Chabierski et al. [24]. The expression levels were very high to low (Table 1 and Fig. 1).

**Table 1:** An overview of used E-protein derived peptides and their characteristics.

Peptide n°	E-proteinderivedpeptides	MHCclass I	MHCclass II	Expression	Domain withinWNV E protein	Sequences ofknown humanT cell epitopewithin our peptides	References
15	M66-75/E1-20			−	I		
16	E11-40			++	I		
17	E31-60		x	−	I/II		
18	E51-80	x	x	+	II		
19	E71-100			++	II		
20	E91-120	x		++	II	RGWGNGCGLFGKGSI	[30], [31]
21	E111-140	x		++	II/I		
22	E131-160		x	−	I		
23	E151-180	x	x	++	I		
24	E171-200			++	I		
25	E191-220		x	+	I/II		
26	E211-240		x	+	II	TFLVHREWFMDLNLPW	[13], [32]
27	E231-260		x	++	II		
28	E251-280	x	x	+	II		
29	E271-300		x	−	II/I		
30	E291-320	x		+	I/III	EKLQLKGTTYGVCSKAFK	[30]
31	E311-340	x		+	III		
32	E331-360	x		−	III		
33	E351-380			+/−	III		
34	E371-400	x		+	III	KVLIELEPPFGDSYIVV	[31]
35	E391-420	x		+	III	HKSGSSIGKAFTTTLKGA	[31]
36	E411-440	x	x	−	III	SVGGVFTSV	[33], [34], [35]
						WDFGSVGGVFTSVGKAVH	[13]
37	E431-460	x		+	III	FRSLFGGMSWITQGLLGA	[30]
38	E451-480	x		+	III	FRSLFGGMSWITQGLLGA	[30], [31]
39	E471-500	x	x	+/−	III		
40	E491-501/NS11-18			+	III		

Amino acid sequences of the E-protein derived peptides used in this study, their expression level in *E. coli*, and the presence of predicted MHC class I or class II epitopes in the different domains of the E-protein compared to known human T-cell epitopes. The sequence of the peptides can be found in Chabierski et al. [24].

Abbreviations. E: WNV envelope protein, M: WNV membrane protein, NS: WNV non-structural protein ++: very high expression, +: high expression, +/−: moderate expression and –: very low expression.

From six clones the amount of protein obtained was not sufficient to be used as stimulant in ELISPOT assays. To expand the WNV-E specific T-cell repertoire, five BALB/c mice were intramuscularly immunized (two vaccinations with four-weeks interval) followed by *in vivo* electroporation (BTX ECM 830, Harvard apparatus, Holliston, MA, USA) of a plasmid encoding the prM/E expression cassette [23]. This plasmid was prepared by cloning the prM/E expression cassette from pCBWN (kindly provided by Mike Diamond, Washington University) into the pVAX1 (Invitrogen) backbone. Five untreated BALB/c mice served as naive control mice. The ethics committee of the faculty of Veterinary Medicine of Ghent University authorized the animal experiments (permit EC2012/124). Two weeks after the boost, blood was collected and splenocytes were isolated. Splenocyte suspensions were depleted of red blood cells using ammonium chloride hypotonic lysis and passed through a m cell strainer. To obtain enough splenocytes for CD4+ and CD8+ isolation, we pooled the splenocytes of the vaccinated as well as the naive control mice, and subsequently divided the pooled cells into two cell preparations. In the first preparation CD8+ T cells were depleted with magnetic CD8 Dynabeads (Life Technologies, Merelbeke, Belgium) and in the other preparation CD4+ cells were depleted with magnetic CD4 Dynabeads (Life Technologies, Merelbeke, Belgium) in accordance with the manufacturer’s recommended protocol. To confirm the depletion, spleen cells were stained with PerCP-anti-CD4 (Biolegend, Uithoorn, The Netherlands) or PerCP anti-CD8 (Biolegend, Uithoorn, The Netherlands) and analyzed by flow cytometry (Accuri C6, BD Biosciences, Erembodegem, Belgium). The extent of depletion obtained was 97% and 93% for respectively CD4− and CD8-depletion. Next, ELISPOT assays were performed to measure the IFN-γ and IL-4 production by CD4 and CD8 depleted splenocytes. In detail, sterile 96-well Maxisorp immuno-plates were coated with anti-IL-4 (Biolegend, Uithoorn, The Netherlands) or anti-IFN-γ monoclonal antibodies (Biolegend, Uithoorn, The Netherlands) and blocked with sterile PBS containing 1% BSA for 1 h at 37°C. Next, 3×10^5^ CD8− or CD4− depleted splenocytes were plated in 100 µl of culture medium and stimulated during 16h with culture medium with GST (negative control), g/mL Concanavalin A (ConA, positive control, Sigma–Aldrich (Diegem, Belgium)), 2 µg/ml purified E-protein [25] or 2 µg/ml of purified recombinant GST-peptides. After stimulation the plates were washed two times with PBS and four times with PBS containing 0,05% Tween-20. IL-4 or IFN-γ trapped on the plates was detected by a biotinylated monoclonal anti-IL-4 (Biolegend, Uithoorn, The Netherlands) or anti-IFN-γ antibody (Biolegend, Uithoorn, The Netherlands). Subsequent incubation with GABA-conjugated streptavidin (U-Cytech Biosciences, Utrecht, The Netherlands) was used to develop silver spots at places where immune cells secreted IL-4 or IFN-γ during stimulation. Splenocytes from the vaccinated and non-vaccinated mice were analyzed in triplicate and the spots were counted using the Bioreader 5000 (Bio-sys, Karben, Germany). IgG antibody titers were determined as described by Oliphant et al. [25]. Briefly, two weeks after the boost, blood samples were collected by cardiac puncture. Blood was allowed to clot for 60 min at 37°C, and serum was obtained by combining the supernatant from two successive centrifugations. The titers of E-specific IgG1 and IgG2a antibodies in the serum were determined by ELISA in 96-well Maxisorp immuno-plates (Nalge Nunc, Rochester, USA) coated overnight with recombinant E-protein (1 µg/ml in carbonate buffer, 100 µl/well, 4°C). After coating, the plates were washed three times with PBS containing 0.1% Tween-20 and blocked with 3% skim milk in PBS. Next, three-fold serial dilutions of mouse serum, starting with a 1/100 dilution, were incubated for 1 h while shaking at room temperature. After washing goat-derived anti-mouse serum conjugated with horseradish peroxidase specific for mouse isotypes IgG1 or IgG2a (Southern Biotechnology Associates, Birmingham, USA) and tetramethylbenzidine substrate (BD Biosciences) were used to determine specific antibody titers. Antibody titers are defined as the reciprocal of the highest dilution with an OD450 that is at least double the OD450 of pre-immune serum samples.

**Figure 2:**
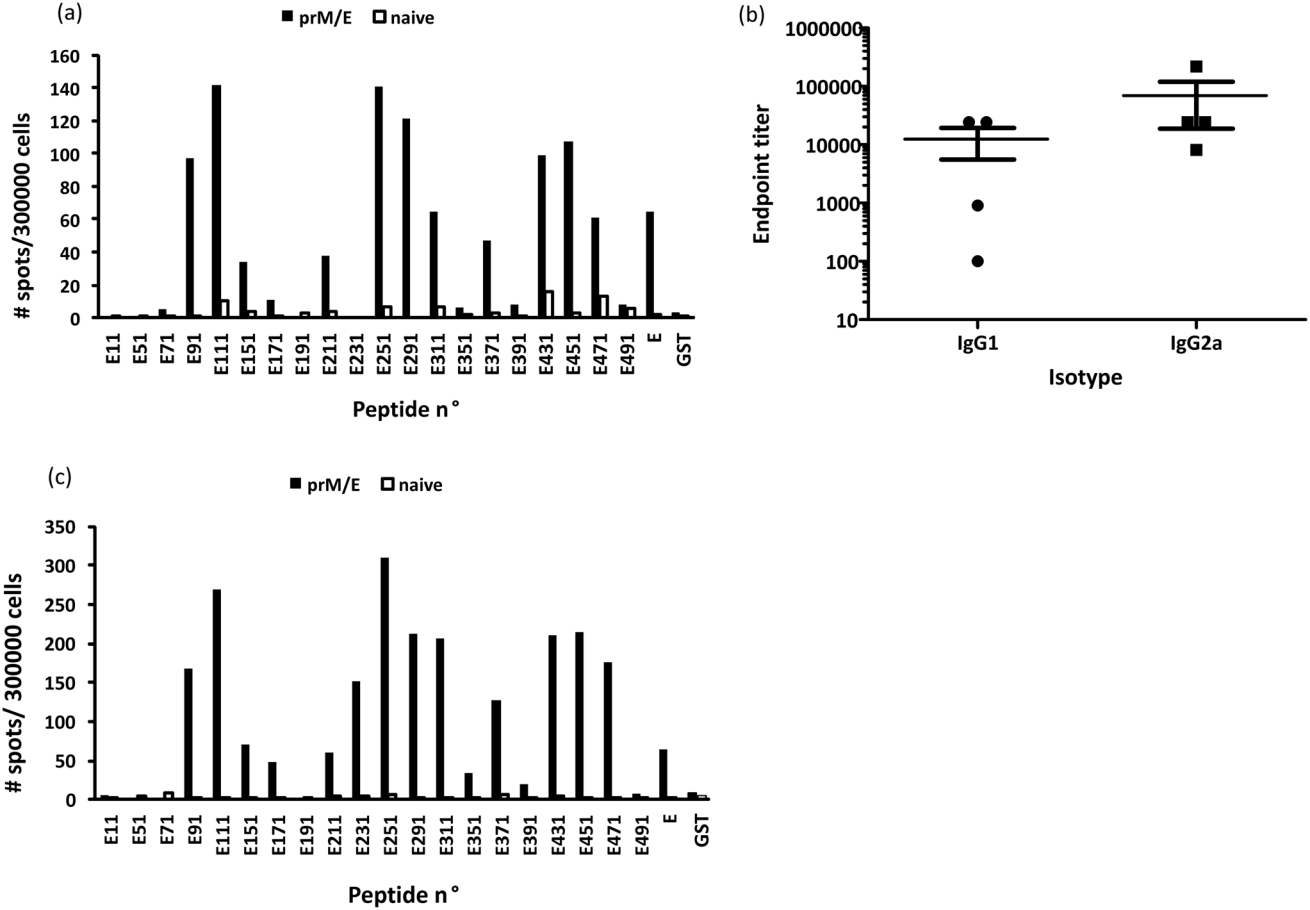
Detection of cellular and humoral immune response following pDNA-based vaccination. IFN-γ production by (a) CD4-depleted and (c) CD8-depleted splenocytes after stimulation with purified recombinant GST tagged E-protein derived peptides. The WNV E-protein specific T-cell repertoire in BALB/c mice was expanded by two DNA vaccinations. Splenocytes obtained two weeks after the boost were stimulated with different recombinant GST tagged E-protein derived peptides and the numbers of cells producing IFN-γ were determined via ELISPOT. (b) Detection of serum IgG1 and IgG2a titers to the WNV E-protein two weeks after the boost via ELISA.

To predict MHC class I and II T cell epitopes, we used the immune epitope database (IEDB) analysis tool (http://www.iedb.org) [22]. The binding affinity of peptides (15-mers) to MHC class II (H2-IEd and H2-IAd) was predicted. In this case, the SMM-align method was employed to find out good MHC class II candidate binders. The top scoring peptides were selected by setting cut-off values of IC50 for the predicted binders at 250 nM. The MHC class I binding predictions were made using the IEDB analysis resource Consensus tool, which combines predictions from ANN aka NetMHC (3.4), SMM and Comblib. All the available MHC alleles were selected and the peptide lengths were set at respectively 9, 10 and 12 for H2-Kd, H2-Dd and H2-Ld before making prediction. Strong binder to selected MHC I alleles were classified based on the binding affinity thresholds ≤50 nM. Overall, this computer driven algorithm predicted that 14 and 11 of the E-protein derived peptides contained epitopes that were specific for respectively MHC class I and II (Table 1). Interestingly, we found a clear difference of the epitope distribution within the different structural domains of the E-protein. Most of the epitopes located in domain III were peptides binding to MHC class I molecules, whereas the majority of the epitopes located in domain II are peptides that preferentially bind to MHC class II molecules (Table 1). Next, we wanted to confirm that the predicted epitopes were indeed recognized by CD8+ or CD4+ T cells. Therefore, we stimulated the CD4− or CD8-depleted splenocytes with the purified E-protein derived peptides and measured the IFN-γ and IL-4secretion via ELISPOT assays. Twenty recombinant polypeptides were tested for their ability to stimulate CD8+ or CD4+ spleen-derived T cells from BALB/c mice immunized against WNV E-protein. As a negative control, splenocytes preparations from naïve mice were used. Out of the 14 predicted MHC class I epitopes we could purify 12 peptides and, 11 were confirmed to be able to induce IFN-γ secretion by CD8+ T cells. However, peptide E391 that was also predicted to stimulate CD8+ cells did not stimulate IFN-γ production. Interestingly, a new epitope (present in peptide E211), for which the MHC class I restriction was not ascertained by the IEDB analysis tool, was identified (Fig. 2a).

**Figure 3:**
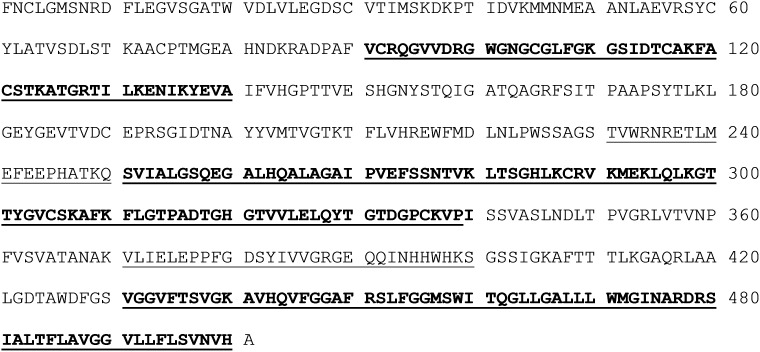
Location of the peptide sequences in the E protein that, based on our *in vivo* experiments, contain strong CD4+ (underlined) and CD8+ (bold) T cell epitopes. The shown amino acid sequence is that of the E protein of lineage 1 WNV strain Ita09. Sequences that are in bold and underlined contain strong CD4+ as well as CD8+ T cell epitopes.

Peptides with atypical anchoring residues for a related MHC-I allele always display very low binding affinity for MHC-I molecule and probably therefore epitope E211 was not picked up by the IEDB analysis tool. However, some of these peptides may possess potent antigenicity to induce robust and specific T cell responses. The absence of an IFN-γ response to the different peptides in naïve mice demonstrates the specificity of the anti-peptide responses observed. Brien et al. identified six CD8 T cell epitopes in C57BL/6 mice by using overlapping WNV 15-mer peptides [14]. We identified more positive peptides most likely due to the fact that we used 30-mer peptides and certain epitopes are present in two peptides like e.g. H2-Dd epitope RSLFGGMSWI that is present in both peptides E431 and E451. Fig. 3 depicts the location of our peptide sequences in the WNV E protein.

Some of our detected peptides match with T-cell epitopes that were identified in WNV-infected humans (e.g. E91, E211, E291 (see also Table 1)). Additionally, some of the peptides that elicited a strong IFN-γ response are known epitopes for neutralizing antibodies or belong to regions of the WNV E-protein that are very important for the virus’s functionality. For example, peptide E91 is part of the fusion loop domain [26], E291 and E311 are located in lateral ridge of domain III, which has been shown to elicit virus-specific neutralizing antibodies [27], and E431 is part of the stem-anchor region that is important in virus-cell membrane fusion [28].

Surprisingly, we could not detect IL-4 responses in the CD4+ splenocytes with any of the twenty purified peptides. Additionally, even when the full-length E protein was used to stimulate the CD4+ cells we did not observe IL-4 production (data not shown). On the other hand, immunized mice were able to raise antibodies to the E-protein and a Th1-type response was noticed since higher levels of IgG2a were obtained compared to the IgG1 levels (Fig. 2b). However, with thirteen of the purified peptides we could detect a robust IFN-γ response after *ex* *vivo* stimulation of the CD4+ splenocytes (Fig. 2c). Only five of them were predicted by the IEDB analysis tools. Two of the predicted peptides (18 and 25) did not stimulate IFN-γ secretion, whereas several new epitopes were identified in the domain III of the E protein (peptides E291, E311, E371, E431). Since still 7% of the CD8+ cells were not depleted in the CD4 splenocytes preparation it is conceivable that some of the peptides detected may be restricted to MHC class I molecules. However, the number of spots were reasonably higher compared to the number of spots obtained in the CD8+ assays. On the other hand, since the peptides are 30 amino acids long it is possible that they contain both CD4 and CD8 epitopes. Indeed, Hughes *et al.* identified in C57/BL/6J mice a strong CD4+ epitope in WNV E between amino acids 466–495 [16] that had previously been shown by Brien *et al.* [14] to contain a potent CD8+ T cell epitope with protective cytotoxic capabilities. Peptide E231 specifically induced IFN-γ production in CD4+ splenocytes but not in CD8+ splenocytes.

One may notice that the number of spots obtained after stimulation of the CD4+ and CD8+ T cells with WNV E protein is lower than after stimulation with the purified GST fusion peptides (Fig. 2a and 2c). This can be explained as follows. For the stimulation of the CD4+ and CD8+ T cells we used an equal “mass/volume” concentration of the E-protein and the purified recombinant GST-peptides. However, the molecular weight of the E-protein (the ectodomain was used in this study) is about twice that of the GST-peptides. Consequently, the molar concentration of the GST-peptides was about twice the molar concentration of the E-protein. Thus, T cells stimulated with the GST-peptides were exposed to a two-fold higher number of epitopes. This explains why the number of spots after stimulation with the E-protein was lower.

To conclude, to our knowledge this is the first study that describes a detailed analysis of WNV E determinants recognized by CD4+ or CD8+ T cells in BALB/c mice. We identified several CD8+ and CD4+ T cell epitopes in BALB/c mice immunized against WNV E-protein by employing overlapping peptides spanning the entire E-protein sequence. The DNA vaccine leads to the expression of subviral particles that resemble WNV. Therefore, we can assume that the identified epitopes will also exist during the course of a WNV infection. Computer-assisted prediction of MHC peptide binding is a useful approach to identify possible CD8+ epitopes, although we demonstrated that the accuracy of these predictions must be verified empirically. Regarding the identification of CD4+ T cell epitopes, the less strict binding requirements and thus the limited predictive value of MHC class II motifs [29] makes this approach less suitable. Only epitopes which are proven to stimulate CD4+ T cells *in vitro* or *in vivo* can be used as potential subunit components for the design of vaccines against WNV. Both the results of the cytokine profile produced by WNV-specific CD4+ T cells and the IgG isotype suggest that the CD4 immune response after DNA vaccination shows a Th1 bias.
